# Osseous Hemangioma of the Inferior Turbinate: A Case Report and Review of Literature

**DOI:** 10.1155/crot/4079990

**Published:** 2026-05-24

**Authors:** Yousef Marzouk, Roy Casiano

**Affiliations:** ^1^ Department of Otolaryngology-Head and Neck Surgery, King Abdulaziz University, Jeddah, Saudi Arabia, kau.edu.sa; ^2^ Department of Otolaryngology-Head and Neck Surgery, University of Miami Miller School of Medicine, Miami, Florida, USA, miami.edu

**Keywords:** inferior turbinate, intraosseous cavernous hemangioma, nasal cavity

## Abstract

Intraosseous hemangiomas account for 1% of all primary bone tumors and most frequently affect the skull base and vertebrae. Rarely are they reported in the nasal cavity. We report a case of a 51‐year‐old male who complained of bilateral nasal obstruction. Nasal endoscopic examination showed a significantly enlarged right inferior turbinate with normal mucosal membranes. A CT scan revealed a well‐defined heterogeneous mass with osseous borders originating from the bony lamella of the right inferior turbinate, with a honeycombed appearance. An en bloc transnasal endoscopic resection of the mass and turbinate was performed, revealing an intraosseous hemangioma of the inferior turbinate with a cavernous pattern. The postoperative period was uneventful with no recurrence. A literature review of intranasal intraosseous hemangiomas revealed 2 other cases resected endoscopically successfully.

## 1. Introduction

Hemangiomas account for about 20% of all benign nasal neoplasms [1]. Usually, they arise from the mucosal soft tissues of the nasal cavity, but rarely, they can originate from bone. Hemangiomas arising from bone are referred to as intraosseous hemangiomas and account for less than 1% of all primary bone tumors in the body [2]. Intraosseous cavernous hemangiomas of the inferior turbinate are extremely rare, with only four cases reported in English literature. We present a case of a patient who presented with a nasal mass who underwent endoscopic endonasal resection of an intraosseous cavernous hemangioma of the inferior turbinate.

## 2. Case Presentation

A 51‐year‐old male presented with bilateral nasal obstruction, postnasal drip, and the need to blow his nose. Intranasal corticosteroids were ineffective. Additionally, the patient had a history of undergoing a balloon dilation of his sinuses for a diagnosis of recurrent sinusitis. The referring physician also performed an attempted resection of a “mass originating from the nasal septum,” resulting in a septal perforation. However, the nasal obstruction persisted, and the patient sought a second opinion. Nasal endoscopy showed an enlarged right inferior turbinate with some synechiae from the anterior tip of the right inferior turbinate to the right caudal to the septal remnant and floor of the nose anteriorly, and a large, well‐healed septal perforation more posteriorly. The rest of the nasal examination was within normal. Computed tomography (CT) scan of the paranasal sinuses was performed and showed a well‐defined heterogeneous mass with osseous borders originating from the bony lamella of the right inferior turbinate, with a honeycombed appearance (Figure 1). There was no evidence of bony erosion or destruction of the surrounding soft tissues. A simple outpatient en bloc endoscopic resection of the mass involving the inferior turbinate was performed under general anesthesia with minimal to no bleeding. Histopathological examination showed bony trabeculae separated by proliferation of large, dilated, and thin‐walled vascular channels (Figure 2). These vascular spaces are lined by a single layer of flattened and bland endothelial cells.

FIGURE 1Computed tomography: (a) coronal view and (b) axial view showing right inferior turbinate bony mass and honeycombed appearance.

**Figure:**
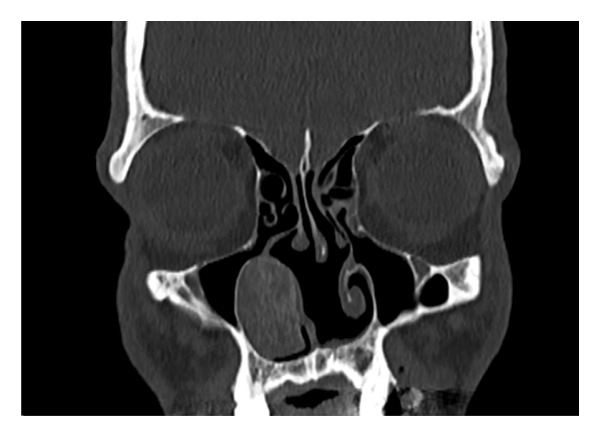
(a)

**Figure:**
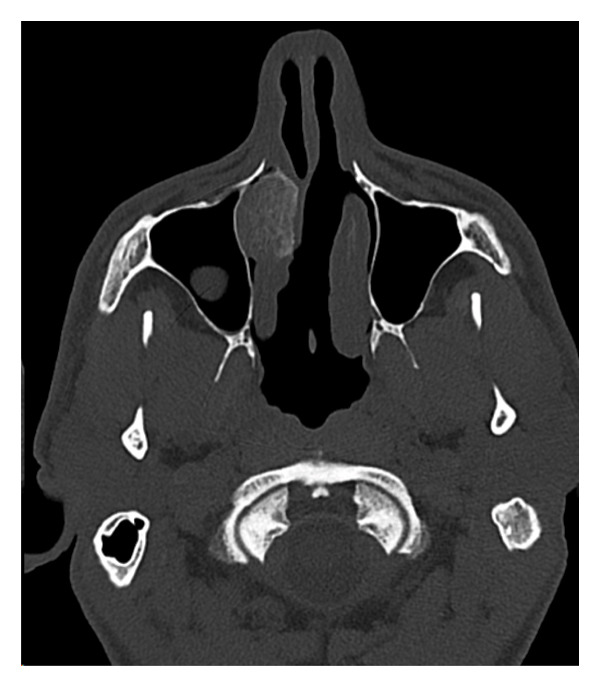
(b)

FIGURE 2Histopathological examination showing blood‐filled, thin‐walled vascular spaces surrounded by bony trabeculae.

**Figure:**
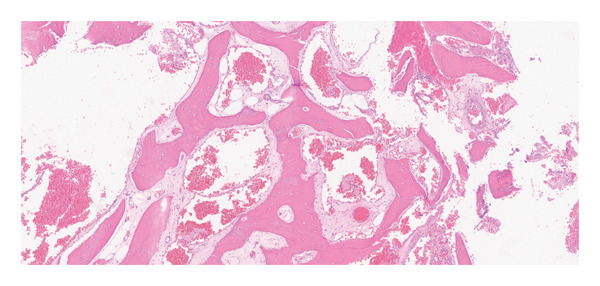
(a)

**Figure:**
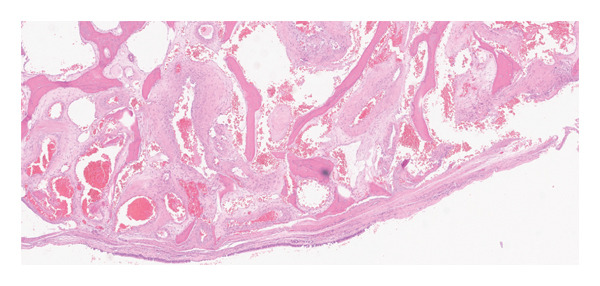
(b)

## 3. Discussion

Hemangiomas are benign vascular tumors and commonly originate in the head and neck area. These types of tumors are twice as common in females as in males and usually present in the fourth and fifth decades of life [3]. The nasal cavity is an uncommon location, with the nasal septum being most frequently affected, followed by the lateral nasal wall and the vestibule [4]. Most are superficial soft lesions of the mucous membranes. Intraosseous hemangiomas are rare and benign tumors that account for 1% of primary bone tumors. The most common site of involvement is the vertebral column [5]. Intraosseous cavernous hemangiomas of the nose are very rare, with the inferior turbinate being reported in only four cases in English literature. A review of all reported cases of intraosseous hemangioma in the nasal cavity in the English literature with their surgical approaches is summarized in Table 1.

**TABLE 1:** Reported cases of intranasal intraosseous hemangiomas.

Author	Sex	Age	Presenting symptoms	Location	Histopathology type	Surgical approach	Outcome
Fahmy et al. [6]	Male	25	Nasal obstruction, sneezing, and rhinorrhea	Inferior turbinate	Cavernous	Midfacial degloving approach	No recurrence
Takeda et al. [7]	Female	73	Nasal obstruction	Inferior turbinate	Cavernous	Caldwell‐Luc procedure	No recurrence
Akiner et al. [3]	Female	57	Nasal obstruction	Inferior turbinate	Cavernous	EEA^∗^	No recurrence
Akiyama et al. [5]	Female	56	Incidental finding of right maxillary opacification when patient went to dental clinic	Middle turbinate	Cavernous	EEA	No postoperative complications
Kim et al. [8]	Male	52	Incidental finding of nasal cavity mass on MRI for headache	Middle turbinate	Cavernous	EEA	No recurrence
Kim et al. [9]	Male	64	Nasal obstruction	Middle turbinate	Cavernous	EEA with partial middle turbinectomy	No recurrence
Azreen Zaira et al. [10]	Male	55	Epistaxis, nasal obstruction	Crista Galli with extension into left nasal cavity	Cavernous	EEA	No recurrence
Goomany et al. [4]	Female	47	Nasal obstruction	Inferior turbinate	Cavernous	EEA	No recurrence
Caceres et al. [11]	Male	70	Nasal congestion, postnasal drip, rhinorrhea, and hyposmia	Anterior ethmoid sinus	Cavernous	EEA with mass excision	No recurrence

^∗^EEA: endoscopic endonasal approach.

Hemangiomas are subclassified according to the histological appearance into cavernous, capillary, and mixed types. Cavernous hemangiomas are composed of large blood‐filled vascular spaces lined with endothelium. In contrast, the capillary type has small blood vessels lined with epithelium, and the mixed type demonstrates varying sizes of thin‐walled blood vessels [1, 4]. Intraosseous hemangioma usually shows a cavernous pattern, while capillary and mixed configurations are less common [11].

The cause of intraosseous hemangioma is not known, but local trauma is a possible contributing factor [7]. The patient reported in this case report did not report a history of local trauma but had a history of balloon dilation of the maxillary sinus on the ipsilateral side of the tumor which could have been causing symptoms mistakenly attributed to possible chronic maxillary sinusitis. However, there were no previous scans available, and at the time of surgery, the maxillary sinus was normal on CT scan. Therefore, it is unlikely that balloon dilation of the maxillary sinus was an inciting factor in this case.

A CT scan is the diagnostic imaging of choice to diagnose cavernous hemangiomas. The characteristic findings include the presence of a honeycombed appearance, with a sunburst pattern of trabeculations and soap‐bubble configuration [12]. Other imaging modalities that may be useful are MRI and angiography. The latter shows the increased vascularity associated with these tumors.

There are different therapeutic options available to treat cavernous hemangiomas of the nasal cavity such as surgery, radiotherapy, and embolization. Radiation is not commonly used due to being a benign neoplasm, as well as radioresistance and its potential long‐term carcinogenic effects particularly in younger patients [13]. The mainstay of treatment is complete surgical excision with a margin of overlying mucosa and bone [7]. Arterial embolization and sclerotherapy are other treatment modalities, but these are reserved for palliative cases or extremely large lesions with a significant vascular supply [3, 14]. Preoperative embolization has been shown to reduce the risk of intraoperative bleeding in patients with perioral hemangioma, for example, [2]. However, there is no clear evidence that preoperative embolization should be used routinely in most cases, as it is difficult to identify an exact vascular supply and the potential risks of embolization outweigh the benefits [8, 12]. None of the cases of inferior turbinate cavernous hemangioma reported in the literature were associated with any significant intraoperative bleeding [3, 4, 6, 7].

All the previously reported cases of nasal intraosseous cavernous hemangioma were treated using an endoscopic endonasal approach (EEA), except for two patients in whom a Caldwell‐Luc or midfacial degloving approach was performed, respectively [6, 7]. None of them had postoperative complications, and there was no evidence of recurrence on follow‐up.

## 4. Conclusion

We report an extremely rare case of an intranasal intraosseous hemangioma presenting as a submucosal bony mass of the inferior turbinate. A CT scan is diagnostic and reveals its intraosseous nature without destructive findings of the adjacent soft tissues or bone. Local endonasal resection represents the treatment of choice.

## Funding

No funding was received for this manuscript.

## Consent

Patient consent was obtained.

## Conflicts of Interest

The authors declare no conflicts of interest.

## Data Availability

The data that support the findings of this study are available from the corresponding author upon reasonable request.
